# Supplementary material for: Reversible single crystal-to-single crystal double [2+2] cycloaddition induces multifunctional photo-mechano-electrochemical properties in framework materials
Source: Nat Commun. 2020 Jun 4;11:2808. doi: 10.1038/s41467-020-15510-7 (PMC7272394; doi:10.1038/s41467-020-15510-7)
Supplement: Supplementary file 1 — Supplementary Information [file 41467_2020_15510_MOESM1_ESM.pdf]

Supplementary Information for

Reversible Single Crystal-to-Single Crystal Double [2+2] Cycloaddition Induces Multifunctional Photo-Mechano-Electrochemical Properties in Framework Materials

Dylan A. Sherman,[a] Ryuichi Murase,[a] Samuel G. Duyker,[a,b] Qinyi Gu,[a] William Lewis,[a,b] Teng Lu,[b] Yun Liu[b] and Deanna M. D'Alessandro[a]*

[a] School of Chemistry, The University of Sydney, Sydney, New South Wales 2006, Australia

[b] Sydney Analytical, Core Research Facilities, The University of Sydney, Sydney, New South Wales 2006, Australia

[c] Research School of Chemistry, The Australian National University, Canberra 2601, Australia

*Corresponding Author: deanna.dalessandro@sydney.edu.au, phone +61 2 9351 3777, fax: +61 3 9351 3329

Experimental Methods:

Supplementary Methods

Single Crystal X-ray Diffraction. Measurements of single crystal X-ray diffraction data for **1** and **4** were undertaken on an Oxford Supernova diffractometer with Cu–*Kα* radiation at 100 K. Diffraction data for **2** was collected on the MX1 beamline at the Australian Synchrotron with 0.71073Å radiation at 100 K. Diffraction data for **3** was collected on the Bruker ApexII FR591 diffractometer with Mo–*Kα* radiation at 100 K. Crystals were extracted from the mother liquor into paratone oil and mounted onto the goniometer for data collection. In the case of **1**, the crystals were cold-mounted under dry ice to prevent the degradation of the crystals. The structures were solved using SHELXT[1] and refined using a full-matrix least squares procedure based upon F^2 using SHELXL.[2] Structure solution and refinement was performed within the WinGX[3] system of programs and OLEX2.[4] Crystal information and details relating to the structural refinements are presented in Table S1. Additional details concerning the crystallographic structure description are also provided below. Data are available from the Cambridge Crystallographic Data Centre as numbers CCDC 1898207-1898210 (**1-4**, respectively).

Powder X-ray Diffraction. Powdered samples were loaded in a 0.5 mm diameter capillary and sealed. For **1**, the sample was loaded into the capillary as a slurry in DMF. Single point measurements were performed over the 5–50° 2θ range with a 0.02° step size and 2° min^{-1} scan rate on a PANalytical X'Pert Pro diffractometer fitted with a solid-state PIXcel detector (40 kV, 30 mA, 1° divergence and anti-scatter slits, and 0.3 mm receiver and detector slits using Cu-Kα (λ = 1.5406 Å) radiation.

Light Irradiated Powder X-ray Diffraction. Light-irradiated PXRD analysis was undertaken on a PANalytical X'Pert Pro diffractometer fitted with a solid-state PIXcel detector (40 kV, 30 mA, 1° divergence and anti-scatter slits, and 0.3 mm receiver and detector slits using Cu-Kα (λ = 1.5406 Å) radiation. A powdered sample of **1** in a slurry of DMF was loaded into a 0.3 mm diameter capillary and 4 x 3 blue LED (12 x 0.28 W) strips were mounted around the capillary. Single point measurements were taken at 30 minute intervals with continuous light irradiation onto the sample.

Electrochemistry and Spectroscopy. Cyclic voltammograms were collected on a BASi Epsilon electrochemical analyser. All measurements were recorded in 0.1 M $[(n\text{-}C_4H_9)_4N]PF_6/CH_3CN$ electrolyte using a glassy carbon electrode, platinum counter electrode and an Ag reference electrode. All potentials are reported in volts versus Fc/Fc^+ couple. UV-Vis-NIR spectra were collected on an Agilent CARY5000 Spectrometer with a Harrick Omni-Diff probe. Spectra were collected between 5000–25000 cm^{-1} and are reported as the Kubelka–Munk transform, where $F(R) = (1-R)^2/2R$.

Solid-State Electrochemistry. Solid state electrochemical measurements were performed using a Basi Epsilon electrochemical analyser. Argon was bubbled through solutions of 0.1 M $[(n\text{-}C_4H_9)_4N]PF_6/CH_3CN$. The CVs were recorded using a glassy carbon working electrode (1.5 mm diameter), a platinum wire auxiliary

electrode and an Ag reference electrode. The sample was mounted on the glassy carbon working electrode by dipping the electrode into a paste made of the powder sample in the supporting electrolyte.

Solid-State UV-Vis-NIR Spectroscopy. UV-Vis-NIR spectra were obtained on powdered samples at room temperature using an Agilent CARY5000 Spectrophotometer equipped with a Harrick Omni-Diff Probe accessory over the wavenumber range 5000–25000 cm^{-1}. Spectra are reported as the Kubelka–Munk transform, where $F(R) = (1-R)^2/2R$.

Solid-State Vis-NIR Spectroelectrochemistry.[3] In the solid state, the diffuse reflectance spectra of the electrogenerated species were collected *in situ* in a 0.1 M $[(n\text{-}C_4H_9)_4N]PF_6/CH_3CN$ electrolyte over the range 5000–25000 cm^{-1} using a Harrick Omni Diff Probe attachment and a custom built solid state spectroelectrochemical cell. The cell consisted of a Pt wire counter electrode and an Ag/AgCl reference electrode in 3 M NaCl aqueous solution. The solid sample was immobilised onto a 0.1 mm thick indium tin oxide (ITO) coated glass slide (which acted as the working electrode) using a thin strip of Teflon tape. The applied potential was controlled using an eDAQ e-corder 410 potentiostat. Continuous scans of the sample were taken on the CARY5000 spectrometer and the potential increased gradually until a change in the spectrum was observed. Spectra are reported as the Kubelka–Munk transform, where $F(R) = (1-R)2/2R$.

Raman Spectroscopy. Single point Raman spectra were measured using an inVia Renishaw Confocal Raman microscope. The laser (785 nm) was focused onto the sample using the Raman microscope (×50 magnification). The Raman spectra were recorded over the 100–3200 cm^{-1} range with 10 seconds exposure time and 10% laser power over 1 accumulation.

Light-irradiated Raman Spectroscopy. Light irradiated Raman spectra were measured using an inVia Qontor Confocal Raman microscope. The laser (785 nm) was focused onto freshly prepared crystals of **1** which were wetted with DMF using the sample stage microscope (×50 magnification). The Raman spectra were recorded over the 600–1700 cm^{-1} range with 1 second exposure time and 5% laser power over 30 accumulations. The light power of the white light from the microscope was measured by a light meter and was 12.45 kFc (=200.4 watts or 134.0 kLumens). Spectra were collected between 30 seconds intervals of light irradiation. The light from the sample stage cavity and room were turned off to ensure no other source of light was irradiating the sample during the experiment.

Isothermal Raman Spectroscopy. Isothermal Raman spectra were measured using an inVia Qontor Confocal Raman microscope. The laser (785 nm) was focused onto single crystals of **3** using the sample stage microscope (×50 magnification). Details of the data range, exposure time, laser power and number of accumulations are identical to those reported above. The samples were placed onto a Linkam Scientific FTIR 600 heating stage and the temperature was set by an external controller. Upon heating the sample, each spectrum was collected in 30 second intervals.

Quantification of the structural transformation. The percentage conversion of framework from **1** to **3** was calculated by monitoring the peak centred at 1527 cm^{-1} in in the light irradiated Raman spectra. The conversion to **3** was deemed complete when full recession or no further change in the 1527 cm^{-1} peak was observed. Deconvolution of the peaks yielded the integrated area which was then used to calculate the percentage conversion as a fraction r relative to the starting spectrum of **1**. Spectral deconvolutions were undertaken in OriginPro software using the Lorentzian model with fixed baselines. The modelled peak positions for each spectrum were adjusted to give the best possible fit with the maximum deviation being 4 cm^{-1}.

The same procedure was used to quantify the conversion of **3** to **1** from the isothermal Raman experiment. In this case,case the percentage conversion was calculated as a fraction relative to the spectrum of **3**. A summary of the parameters is provided in tables S4-5.

Atomic Force Microscopy (AFM). The *In-situ* morphology mapping was conducted by the Asylum Research Cypher Atomic Force Microscopy (Cypher AFM) equipped with the droplet liquid cell. A single crystal sample of **1** was placed on the flat Si-substrate and a drop (~100 μL) of DMF-EtOH was used to immerse the single crystal for preventing the decomposition. The AFM tip (Olympus AC240TM) with the calibrated spring constant, k = 2.07 N/m (resonance peak ~ 70 kHz in the air while ~ 10 kHz in the DMF), was used to conduct the non-contact imaging under the illumination induced by the built-in optical microscopy.

DFT Computational Calculations. DFT calculations on the Py_2TTF dimer in its cofacial and cyclized forms were performed using DMol3 within Materials Studio. Geometry optimisations and energy calculations were carried out using the 'fine' overall quality setting, with the Perdew–Wang (PWC) local density approximation (LDA) functional,[4] Ortmann, Bechstedt and Schmidt (OBS) correction for dispersion forces, and a smearing value of 0.005 Hartrees used to treat partial orbital occupancies. The geometry of the cyclised dimer was optimized while the cofacial geometry was fixed to that determined in the single crystal structure. HOMO and LUMO energies and wavefunctions were calculated, as well as three-dimensional maps of electron density and electrostatic potential.

Additional vibrational frequency calculations were performed using Gaussian 16.[5] The M05-2X functional and 6-311+G(2df,p) basis set were used. Geometries of both the cyclised dimer and individual neutral Py_2TTF ligand were optimized, with the former adopting a conformation close to that found in the crystal structure. The ligand optimized to a non-planar geometry with bending at the sulfur atoms, consistent with the unbound, neutral molecule.

Materials and Syntheses

2,6-Bis(4'-pyridyl)-tetrathiafulvalene (Py_2TTF) was synthesised according to a modified literature procedure as outlined below.[6] $Cd(NO_3)_2\cdot 4H_2O$ and biphenyl-4,4'-dicaryboxylic acid (bpdc) were purchased from Sigma-Aldrich and used without further purification. All solvents were of reagent grade or higher and used without further purification unless otherwise stated. Acetonitrile was distilled from CaH_2 and degassed prior to use. $[(n\text{-}C_4H_9)_4N]PF_6$ was recrystallized three times from EtOH prior to use. ^1H NMR spectra were recorded on a Bruker AVANCE 300 spectrometer operating at 300 MHz. ^1H NMR chemical shifts were referenced against an internal residual solvent resonance. All spectra were recorded at room temperature and are reported in ppm (±0.05 Hz). All deuterated solvents were obtained from Cambridge Stable Isotopes. C, H and N microanalyses were carried out at the Chemical Analysis Facility in the department of Chemistry and Biomolecular Science at Macquarie University, Australia.

Experimental details for single crystal and powder X-ray diffraction, FTIR (ATR), diffuse reflectance, spectroelectrochemical Vis-NIR and electrochemical methods can be found in the supplementary information. Structural refinement details for **1**, **2**, **3** and **4** are provided in table S1 in the supplementary information.

Synthesis of 2,6-bis(4'-pyridyl)-tetrathiafulvalene (Py_2TTF). The synthesis was an adaption of the literature methodology.[6] A reaction scheme is provided in Supplementary Scheme 1.

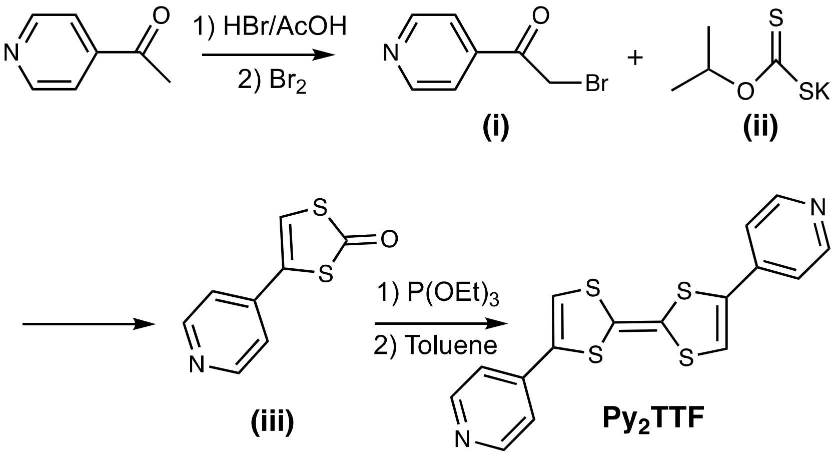

Supplementary Scheme 1. Synthesis of Py_2TTF. 4-Acetyl pyridine (top left), (i) is 4-Bromoacetylpyridine, (ii) is K-*O*-isopropyl xanthate, (iii) is 4-(Pyridine-4-yl)-1,3-dithiol-2one and Py_2TTF (bottom right).

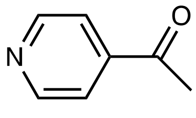

(i) **4-Bromoacetylpyridine.** 4-Acetyl pyridine (15 mL, 0.136 mol) and acetic acid (150 mL, 2.625 mol) were cooled to 0 °C. HBr (5 mL, 48 %) was then added and the mixture allowed to stir for 15 min. Br_2/CH_3COOH

(5 mL, 5 mL) was then added dropwise and the resulting solution stirred at 0 °C for 20 min, followed by stirring at room temperature for 1 h. The formed precipitate (orange) was collected by vacuum filtration, washed with diethyl ether, and heated at 80 ° C to remove excess Br_2 for 1 h to yield 4-bromoacetylpyridine as a white microcrystalline powder (46.7 g, 86.5 %).

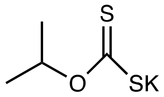

(ii) **K-*O*-isopropyl xanthate.** KOH (26 g, 0.46 mol) was dissolved in isopropanol (300 mL, 3.945 mol) to which CS_2 was added (50 mL, 0.828 mol) at 0 °C. The resulting mixture was stirred for 1 h and brought to room temperature. The mixture was filtered and the resulting product recrystallised from EtOH to yield K-*O*-isopropyl xanthate (55 g, 58.1 %).

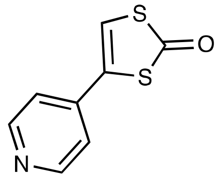

(iii) **4-(Pyridine-4-yl)-1,3-dithiol-2one.** To a suspension of 4-bromoacetylpyridine (22.74 g, 0.113 mol) in dichloromethane (180 mL), triethylamine (13.6 mL, 0.094 mol) was added dropwise followed by the addition of K-*O*-isopropyl xanthate (16.7 g, 0.096 mol). The solution was stirred for 4 h and turned dark red. The resulting mixture was washed with H_2O (2 x 100 mL) and the organic layer isolated. After further washing (H_2O, 6 x 100 mL) the isolated organic layer was heated to remove solvent producing a dark red oil. The oil was dried under N_2, affording a black solid to which H_2SO_4 (98 %, 11.5 mL) was added dropwise. After 10 min, ice was added until the reaction flask was cool. The mixture was neutralised with $NaHCO_3$ (100 mL, 0.1 M) and the precipitate formed was extracted with DCM (3 x 50 mL). The DCM solution was washed with H_2O (6 x 100 mL), solvent was evaporated under vacuum and the remaining product recrystallised from EtOH twice, yielding 4-(pyridine-4-yl)-1,3-dithiol-2one as a bright red solid (3.63 g, 2.2 %).

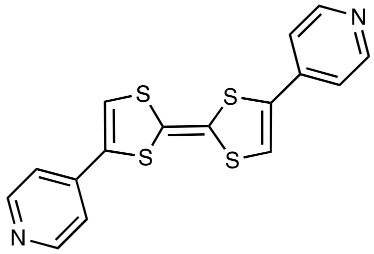

(iv) **Py_2TTF.** 4-(Pyridine-4-yl)-1,3-dithiol-2one (3.63 g, 0.019 mol) was suspended in $P(OEt)_3$ (25 mL, 0.15 mol) and toluene (25 mL) and refluxed under N_2 for 6 h in the absence of light. The resulting product was filtered and washed with MeOH yielding Py_2TTF as a red solid (1.96 g, 18.3 %, m.p. >260 °C). ESI-MS (ESI+, MeOH): *m/z* 357 [M]. ^{1}H NMR (300 MHz, $CDCl_3$): δ 8.65 (d, 2H), 8.61 (d, 2H), 7.31 (d, 2H), 7.13 (d, 2H). 6.88 (s, 2H). ^{13}C NMR (75 MHz, $CDCl_3$): δ 148.5, 144.1; 136.9, 129.1, 120.5. IR (ATR) (cm^{-1}): 2975

(m), 2777 (w), 2456 (w), 2355 (w), 1916 (w), 1584 (s), 1555 (s), 1535 (s), 1403 (m), 1250 (m), 1217 (m), 1166 (w), 1038 (m), 991 (m), 935 (m), 811 (s), 781 (s), 766 (s), 721 (m), 690 (s), 661 (m), 616 (s). Analysis calc. for $C_{16}H_{10}N_2S_4$ (%): C 53.60, H 2.81, N 7.81, S 35.77; Found (%): C 52.74, H 2.98, N 7.54, S 35.12.

Synthesis of [$Cd_2(Py_2TTF)_2(bpdc)_2$]·5DMF (1). $Cd(NO_3)_2·4H_2O$ (11.6 mg, 0.0375 mmol), 2,6-bis(4′-pyridyl)-tetrathiafulvalene (13.5 mg, 0.0375 mmol) and biphenyl-4,4'-dicaryboxylic acid (9.1 mg, 0.0375 mmol) were placed in a 21 mL solvothermal vial which was covered with aluminium foil and dissolved in 4 mL of DMF and 0.75 mL of EtOH. The mixture was heated to 130 ℃ for 10 minutes and further heated at 80 ℃ in a convection oven with no exposure to light, yielding red plate-like crystals after four days. Loss of crystallinity was observed upon exposure to air at room temperature over a period of *ca.* 12 hours, and thus further characterisation of **1** was undertaken on samples wetted with DMF or acetonitrile (MeCN), or over a limited timeframe (< 2 hours) to prevent structural degradation. Yield: 20 mg (30.4 % based on Cd(II)). IR (ATR) (cm^{-1}): 3058 (w), 2928 (w), 2852 (w), 1941 (w), 2468 (w), 1671 (s), 1605 (s), 1576 (s), 1524 (s), 1498 (w), 1393 (s), 1252 (w), 1224 (w), 1176 (m), 1094 (s), 1018 (w), 939 (w), 851 (m), 818 (w), 770 (m), 729 (w), 682 (m), 661 (m), 628 (m). Analysis calc. for $C_{77}H_{81}Cd_2N_9O_{13}S_8$ (%): C 50.46, H 4.48, N 6.92, S 14.08; Found (%): C 50.43, H 4.40, N 6.90, S 14.03.

Synthesis of [$Cd_2(Py_4C_{12}S_8H_4)_{0.84}(Py_2TTF)_{0.32}(bpdc)_2$] (2). Samples of **2** for single crystal X-ray diffraction studies were generated from wetted crystals of **1** in DMF via light irradiation for 6 minutes using white light (25 W) from a microscope lamp. The partially cyclised product was then transferred immediately from the microscope slide to the diffractometer in paratone oil under dark conditions for analysis.

Synthesis of [$Cd_2(Py_4C_{12}S_8H_4)(bpdc)_2$]·3DMF (3). Single crystals of **3** were formed by irradiating single crystals of **1** with white light (25 W) using the beam from a microscope. Bulk **3** was formed by placing a sample of **1** in direct sunlight for 2 days or under a UV lamp (20 W) for 1 h. IR (ATR) (cm^{-1}): 3054 (w), 2918 (w), 2847 (w), 1663 (s), 1605 (m), 1576 (s), 1522 (m), 1384 (s), 1254 (w), 1174 (w), 1090 (m), 1020 (w), 884 (w), 849 (m), 770 (s), 735 (w), 706 (w), 680 (m), 659 (m), 624 (m). Analysis calc. for $C_{71}H_{67}Cd_2N_7O_{11}S_8$ (%): C 50.89, H 4.03, N 5.85, S 15.31; Found (%): C 50.85, H 3.98, N 5.80, S 15.28.

Synthesis of [$Cd_2(Py_2TTF)_2(bdc)_2$]·DMF (4). $Cd(NO_3)_2·4H_2O$ (11.6 mg, 0.0375 mmol), 2,6-bis(4′-pyridyl)-tetrathiafulvalene (13.5 mg, 0.0375 mmol) and 1,4-benzenedicarboxylate (6.4 mg, 0.0375 mmol) were dissolved in DMF (4 mL) and EtOH (0.75 mL). The mixture was sealed and after sonication for 5 min was heated in a solvothermal oven at 80 ℃ for 72 h to yield red plate-like crystals suitable for X-ray diffraction (11.2 mg, 11.3% based on Cd(II)). IR (ATR) (cm^{-1}): 3045 (w), 2930 (w), 2864 (w), 2485 (w), 1195 (w), 1663 (m), 1605 (m), 1549 (s), 1376 (s), 1219 (m), 1090 (s), 1015 (s), 941 (m), 824 (s), 748 (s), 690 (s), 659 (s), 626 (s). Analysis calc. for $Cd_2C_{57}H_{48}N_7O_{12}S_8$ (%): C 45.51, H 3.22, N 6.52, S 17.05; Found (%): C 45.49, H 3.15, N 6.45, S 17.08.

Supplementary Notes

Additional Structural and Crystallographic Details

[$Cd_2(Py_2TTF)_2(bpdc)_2$]·5DMF (1). The structure of **1** possesses an octahedral Cd(II) ion coordinated by two bpdc and two Py_2TTF ligands. Two of these Cd(II) centers form an 8-membered {$(Cd–O–C–O)_2$} ring consisting of two Cd(II) and four bpdc units (Figure S1). The repetition of these secondary building units (SBUs) creates grid-like 2-D sheets of {Cd(bpdc)}$_n$ that propagate in the *a*-*b* direction. While two opposing bpdc ligands coordinated to the Cd dimer SBU remain in-plane with each other, the others bend in opposing directions creating an undulating 2-D sheet (Figure S1). Additionally, a rotation of *ca.* 38° about the central C–C bond between phenyl groups in the bpdc ligand is found, which is likely to accommodate for the puckering in the {Cd(bpdc)}$_n$ sheets. The apical positions of Cd(II) are occupied by Py_2TTF ligands which act to pillar the 2-D sheets to form a 3D framework as shown in Figure S1.

DMF molecules are found in these channels for the as-synthesised material, one of which could be located crystallographically as a half-occupied molecule, while the others were accounted for with PLATON SQUEEZE, giving a formula unit of [$Cd_2(Py_2TTF)_2(bpdc)_2$]·5DMF. This is supported by the thermogravimetric analysis of **1** which exhibits a 16.6% weight loss between 50–130 ºC corresponding to approximately four DMF molecules per formula unit (i.e, the loss of the disordered DMF molecules), but retention of the crystallographically located molecule (Figure S2). Rapid weight loss is observed at 275 ºC upon further heating, indicative of framework decomposition.

[$Cd_2(Py_4C_{12}S_8H_4)(bpdc)_2$]·3DMF (3). Whilst solvent molecules could not be satisfactorily modelled in this structure, the SQUEEZE[7] program in PLATON[8] together with evidence from the elemental analysis and TGA suggest the presence of three DMF molecules per formula unit.

[$Cd_2(Py_2TTF)_2(bdc)_2$]·DMF (4). Single crystal X-ray diffraction revealed **4** to possess the monoclinic space group $P2_1/c$ with unit cell dimensions of a = 19.3285(8) Å, *b* = 19.4632(6)Å, *c* = 17.0969(6) Å and β = 98.710(4)°. PXRD revealed the homogeneity of the bulk product (Figure S6). Examination of the crystal structure revealed **4** to possesses the same {$(Cd–O–C–O)_2$} SBU with Py_2TTF ligands coordinating on the apical position of the Cd(II) ions (Figure S7). Though significant similarity in connectivity exists, **4** was found to possess less undulation in its {$Cd(bdc)_4$}$_n$ sheets when compared with **1** (Figure S7). This is owed to the additional rotational freedom provided by the bdc co-ligand, which helps to alleviate framework strain. Additionally, the shorter bdc ligand gives rise to an almost halving of the void space in framework **4** compared to **1**. A second interpenetrating framework is also found in **4** which exists approximately midway between the first net (Figure S7).

Supplementary Tables

Supplementary Table 1. Data collection and refinement statistics for structures **1-4**. Data are available from the Cambridge Crystallographic Data Centre as numbers CCDC 1898207-1898210 (**1-4**, respectively).

	**1**	**2**	**3**	**4**
Empirical formula	$C_{37.5}H_{35.5}CdN_{4.5}O_{6.5}S_4$	$C_{70.5}H_{57.23}Cd_2N_{7.5}O_{11.5}S_8$	$C_{34.5}H_{28.5}CdN_{3.5}O_{5.5}S_4$	$C_{57}H_{48}N_7O_{12}S_8Cd_2$
Formula weight	893.84	1674.75	820.75	1504.30
Temperature (Kelvin)	100(2)	150(2)	100(2)	150(2)
Wavelength	1.54184Å	0.71073	0.71073	1.54184
Crystal system	monoclinic	monoclinic	monoclinic	monoclinic
Space group	$P2_1/n$	$P2_1/n$	$P2_1/n$	$P2_1/c$
a (Å)	10.1851(4)	9.2500(19)	9.113(2)	19.3285(8)
b (Å)	28.1899(11)	28.297(6)	28.336(7)	19.4632(6)
c (Å)	14.9875(5)	15.864(3)	15.948(4)	17.0969(6)
α (°)	90	90	90	90
β (°)	97.675(3)	95.75(3)	95.629(5)	98.710(4)
γ (°)	90	90	90	90
Volume ($Å^3$)	4264.6(3)	4131.4(15)	4102.8(18)	6357.6(4)
Z	4	2	4	4
ρ_{calcd} (g/cm^3)	1.392	1.346	1.329	1.572
μ (mm^{-1})	6.334	0.774	0.777	8.354
F(000)	1824.0	1697.0	1664.0	3036.0
Crystal size (mm)	0.106×0.089×0.024	0.1×0.02×0.02	0.123×0.11×0.031	0.204×0.155×0.027
Theta range (°)	8.648 to 152.526	3.866 to 64.106	2.872 to 53.016	7.88 to 147.584
Reflections measured	32454	73673	59979	20665
Independent reflections	8813	11671	8412	12312
Completeness to theta	99.76% [74.33°]	97.3% [25.24]	99.9% [25.24]	98.9 [67.684]
Absorption correction	multi-scan	multi-scan	multi-scan	multi-scan
Max. and min. transmission	1.00000, 0.65150	0.4344, 0.3655	0.8620, 0.7368	1.00000, 0.33103
Refinement method	F^2	F^2	F^2	F^2
Data / restraints / parameters	8813/61/426	11671/1383/639	8412/874/482	12312/652/783
Goodness-of-fit on F^2	1.009	1.056	1.198	1.041
Final *R(F)* indices [$I > 2\sigma(I)$]	0.0658	0.0603	0.0958	0.1078
wR2 (F^2) indices (all data)	0.1887	0.1632	0.2328	0.3045
Largest diff. peak and hole ($e/Å^3$)	1.93/-0.79	1.38/-2.06	2.80/-1.11	5.28/-2.00

Supplementary Table 2. Experimental, calculated and vibrational mode assignments of **1**. Vibrational modes (ν) of Py_2TTF and ($Py_4C_{12}S_8H_4$) are shown in Supplementary Scheme 2.

**1** (cm^{-1}) (Experimental)	**1** (cm^{-1}) (Calculated)	**Assignments**
439	482	ν_8 (w), ν_5 (d)
495	504	ν_2 and ν_7 (st), ν_4 (b)
624	634	Pyridyl ring (d)
831	845	ν_3 (st)
847	879	ν_7 (st)
861	902	Pyridyl ring hydrogens (b)
940	970	ν_6 (st), Pyridyl ring (d)
1017	1032	Pyridyl ring (w)
1228	1264	Pyridyl ring hydrogens (s)
1264	1281	ν_6 (b), Pyridyl ring (b)
1287	1301	ν_8 (st), Pyridyl ring (d)
1338	1375	Pyridyl ring (w)
1424	1471	Pyridyl ring (w)
1497	1560	ν_5 (st), Pyridyl ring (s)
1527	1632	ν_1 (st), ν_5 (st), ν_2 (b)
1544	1646	ν_1 (st), Pyridyl ring (d)
1608	1687	ν_1 (st), ν_5 (st), Pyridyl ring (w)

***b = bending, d = deformation, st = stretch, w = wagging, s = scissoring**

Supplementary Table 3. Experimental, calculated and vibrational mode assignments of **1**. Vibrational modes (ν) of Py_2TTF and ($Py_4C_{12}S_8H_4$) are shown in Supplementary Scheme 2.

**3** (cm^{-1}) (Experimental)	**3** (cm^{-1}) (Calculated)	**Assignments**
484	497	ν_1 (b)
503	523	ν_2 (b), ν_3 (w)
542	564	Pyridyl ring (w)
624	630	ν_3 (st), Pyridyl ring (d)
661	688	Pyridyl ring (w)
682	704	ν_5 (st)
735	830	ν_7 (st), Pyridyl ring hydrogens (b)
783	880	ν_3 (st)
848	920	ν_5 (b)
862	934	ν_7 (st)
1015	1033	Pyridyl ring (d)
1078	1121	Pyridyl ring hydrogens (b)
1148	1207	ν_8 (s), ν_7 (st)
1211	1256	ν_8 (s), ν_9 (st)
1229	1269	ν_9 (st), Pyridyl ring (w)
1265	1285	ν_8 (st), ν_9 (st)
1283	1306	ν_3 (st), ν_9 (st)
1411	1563	Pyridyl ring (d)
1527	1662	ν_1 (st)
1559	1685	Pyridyl ring (w)

***b = bending, d = deformation, st = stretch, w = wagging, s = scissoring**

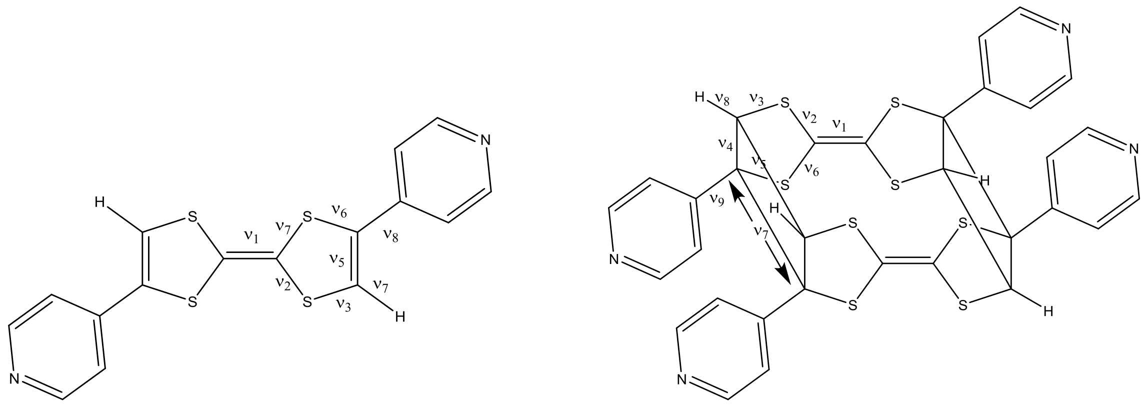

Supplementary Scheme 2. Vibrational modes (ν) of Py_2TTF (left) and ($Py_4C_{12}S_8H_4$) (right).

Supplementary Table 4. Table summarising the quantification of the conversion of **1** to **3** from the photo-irradiated Raman spectra.

**Time (Seconds)**	**Area (Integral of 1527 cm^{-1} peak)**	**Percentage of 3 (%)**
0	279315	0
60	263744	5.57483
120	168088	39.8212
180	146367	47.5976
240	105282	62.3068
300	54392	80.526
360	48226	82.734
420	24236	91.322
480	17775	93.636
540	3872	98.61
600	3845	98.62

Supplementary Table 5. Table summarising the quantification of the retro-conversion of **3** to **1** from the isothermal Raman spectra.

**Time (seconds)**	**Area (Integral of 1527 cm^{-1} peak)**	**Percentage of 1 (%)**
0	0	0
30	424.82	0.14996
60	114094	40.2768
90	181767	64.1662
120	188066	66.3901
150	209726	74.0362
180	205843	72.6655
210	227249	80.2221
240	235654	83.1892
270	283275	100

Supplementary Figures

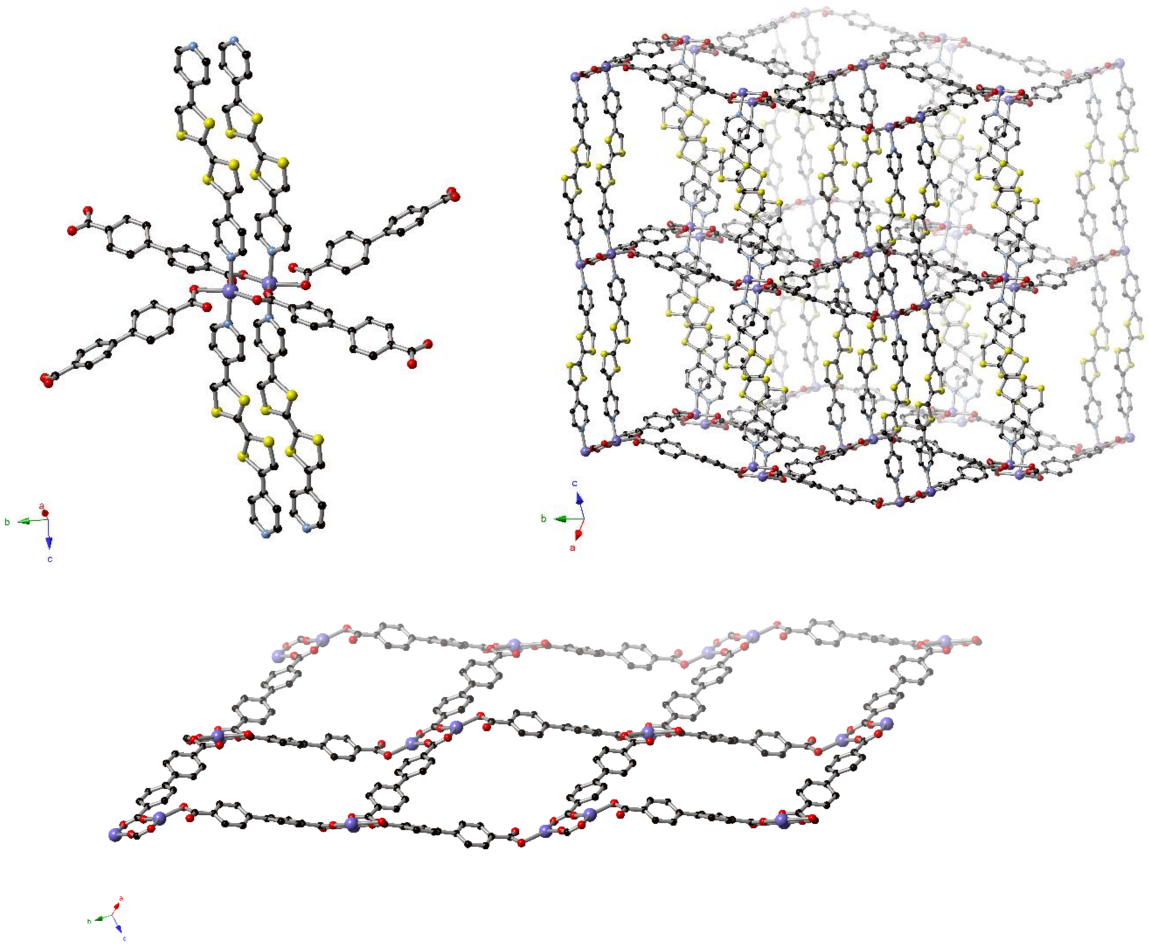

Supplementary Figure 1. Crystal structure of $[Cd_2(Py_2TTF)_2(bpdc)_2]$ showing the SBU (top left), a single net (top right) and undulating sheets of $\{Cd(bpdc)\}_n$ (bottom).

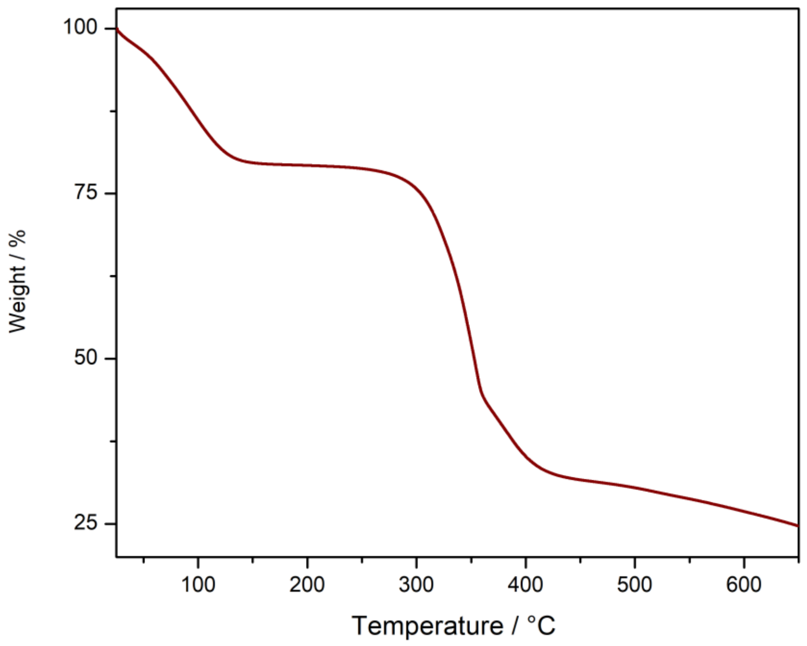

Supplementary Figure 2. TGA of **1** under N_2.

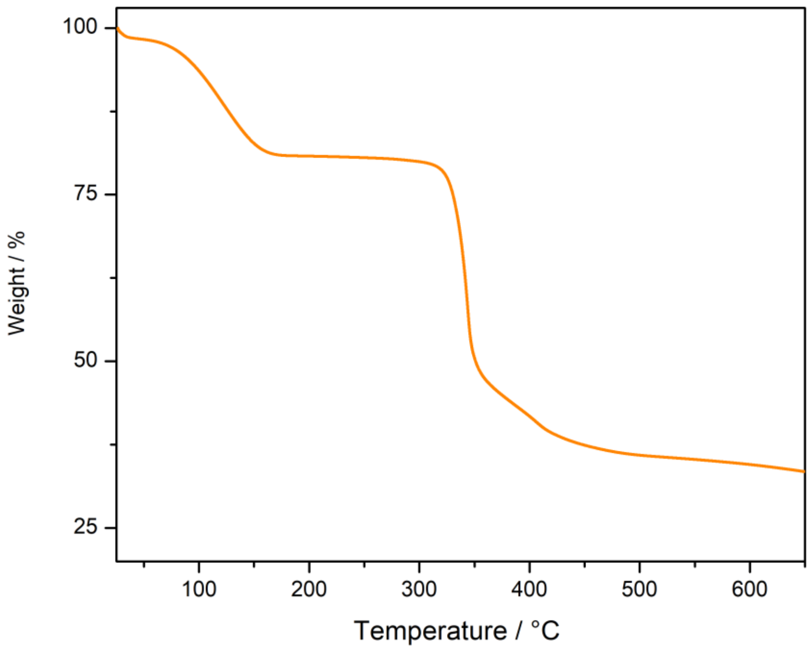

Supplementary Figure 3. TGA of **3** under N_2.

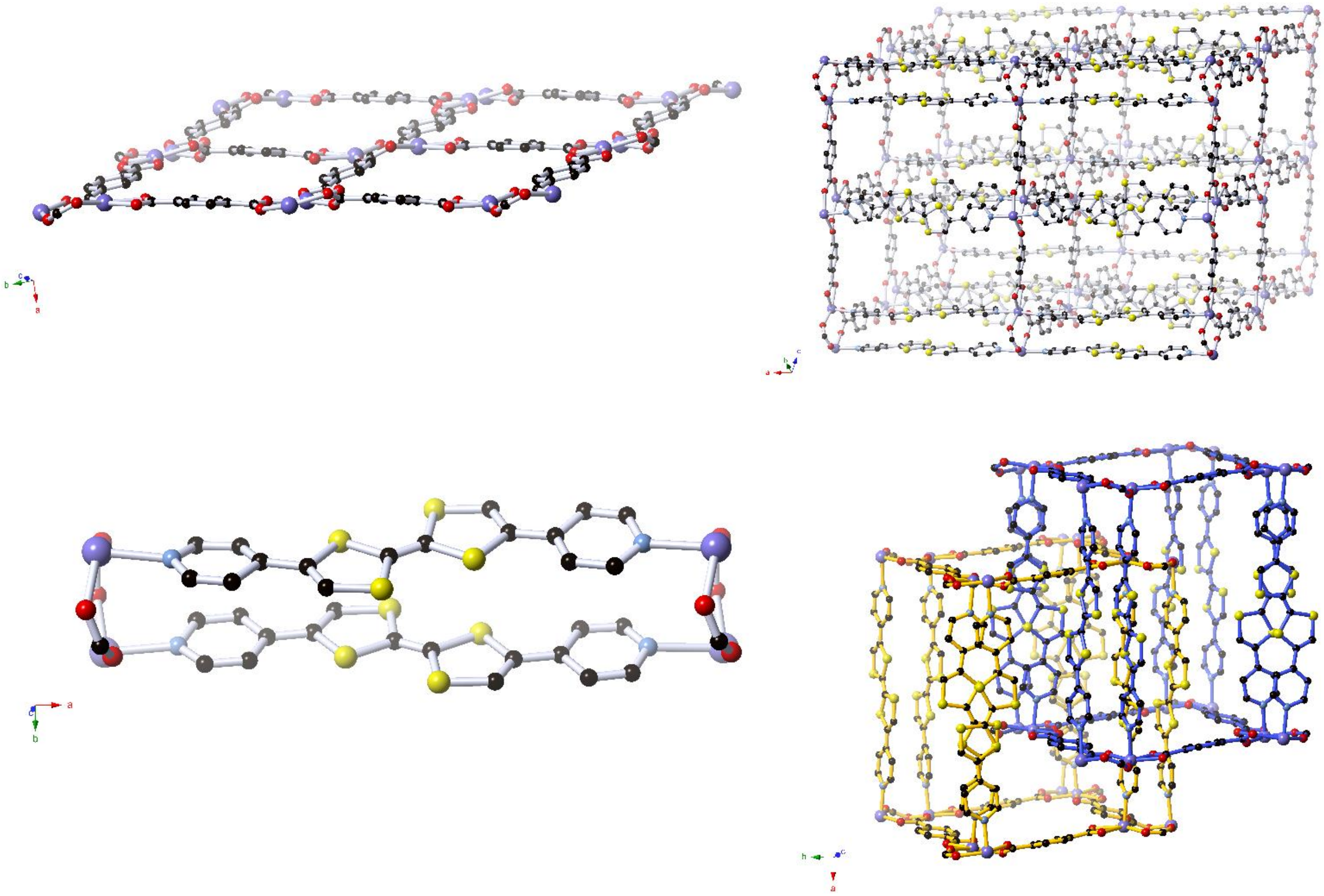

Supplementary Figure 4. Crystal structure of **4** showing {Cd(bdc)}$_n$ sheets (top left), one net of **4** (top right), the cofacial Py_2TTF units (bottom left) and interpenetrated nets of **4** (bottom right). The independent nets have been highlighted in yellow and blue. Solvent and hydrogen molecules have been excluded for clarity.

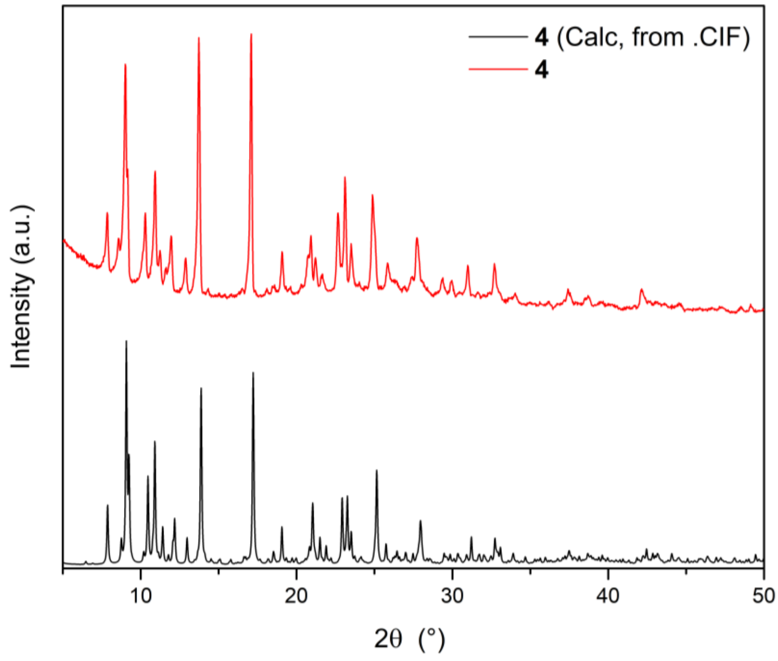

Supplementary Figure 5. PXRD of **4** (red) and calculated pattern (black).

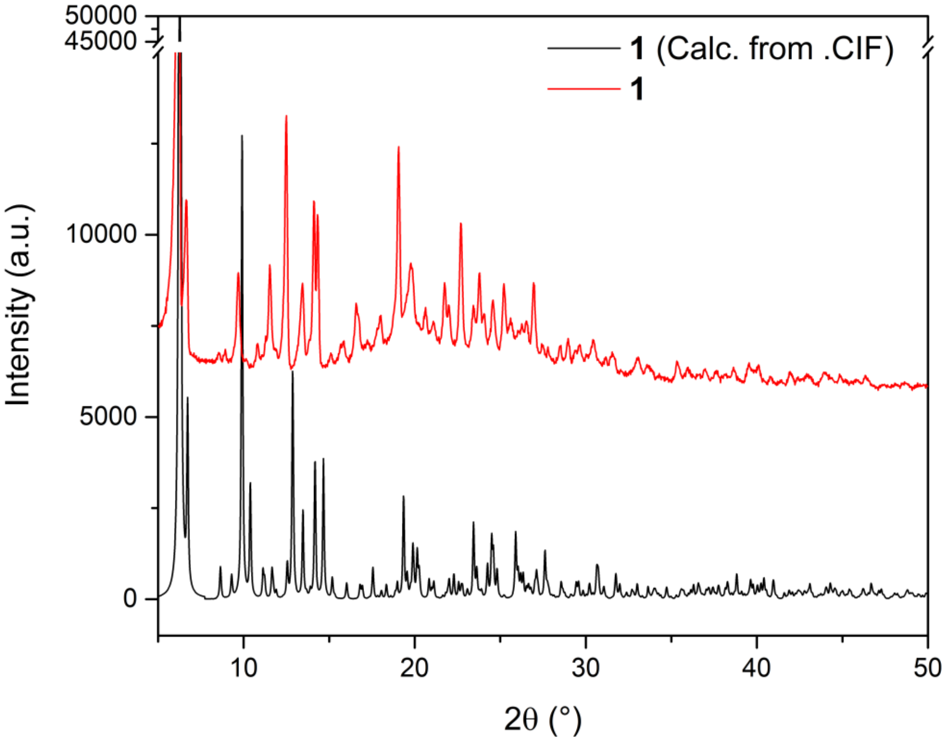

Supplementary Figure 6. PXRD of **1** at 298 K as a slurry in DMF-EtOH and the calculated powder pattern of **1**.

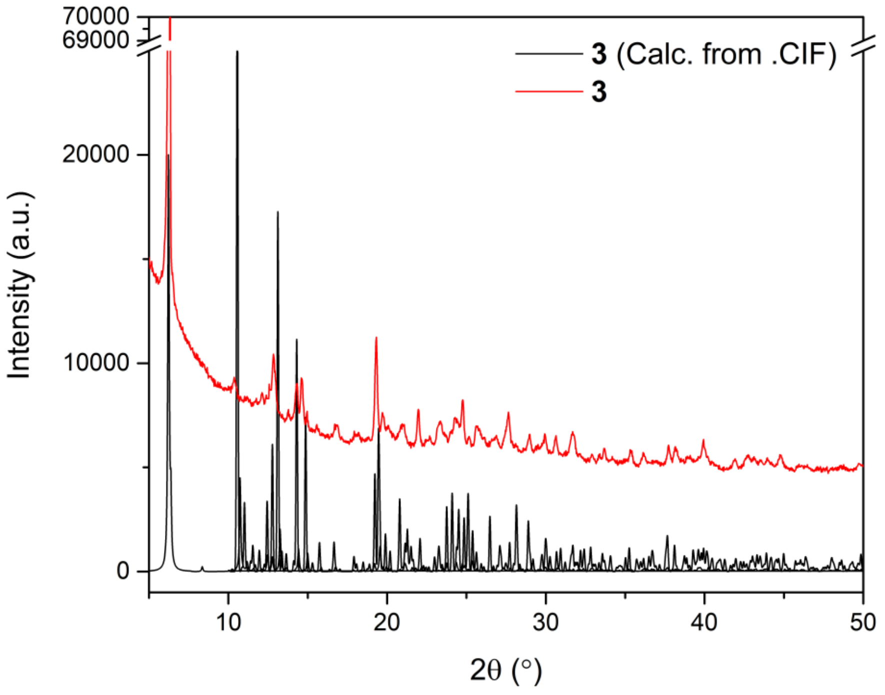

Supplementary Figure 7. PXRD of **3** at 298 K and the calculated powder pattern of **1**.

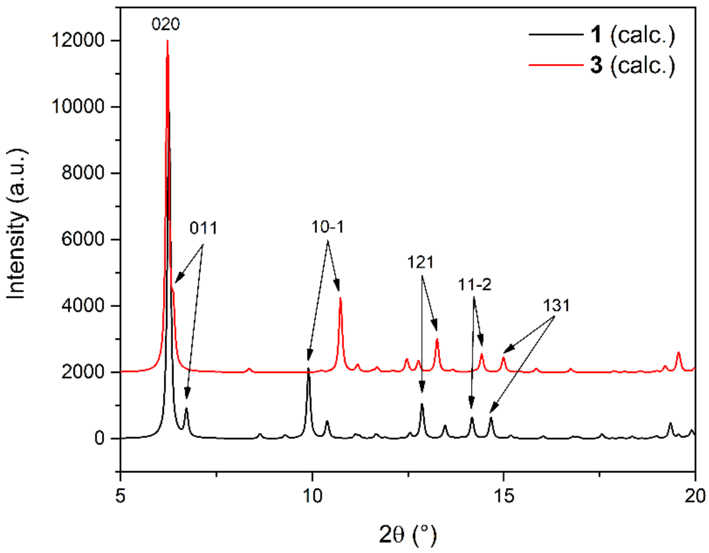

Supplementary Figure 8. Calculated PXRD patterns of **1** (black) and **3** (red) between 2θ = 5–20°. Selected (hkl) indices have been labelled.

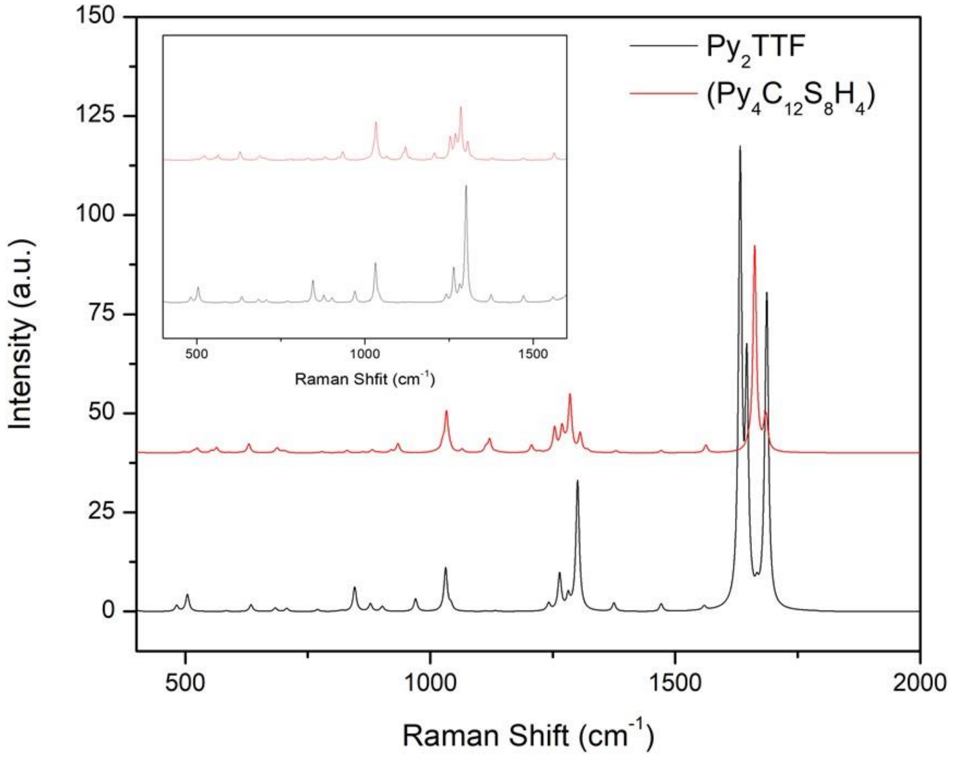

Supplementary Figure 9. Calculated Raman spectra of the Py_2TTF and ($Py_4C_{12}S_8H_4$) fragments.

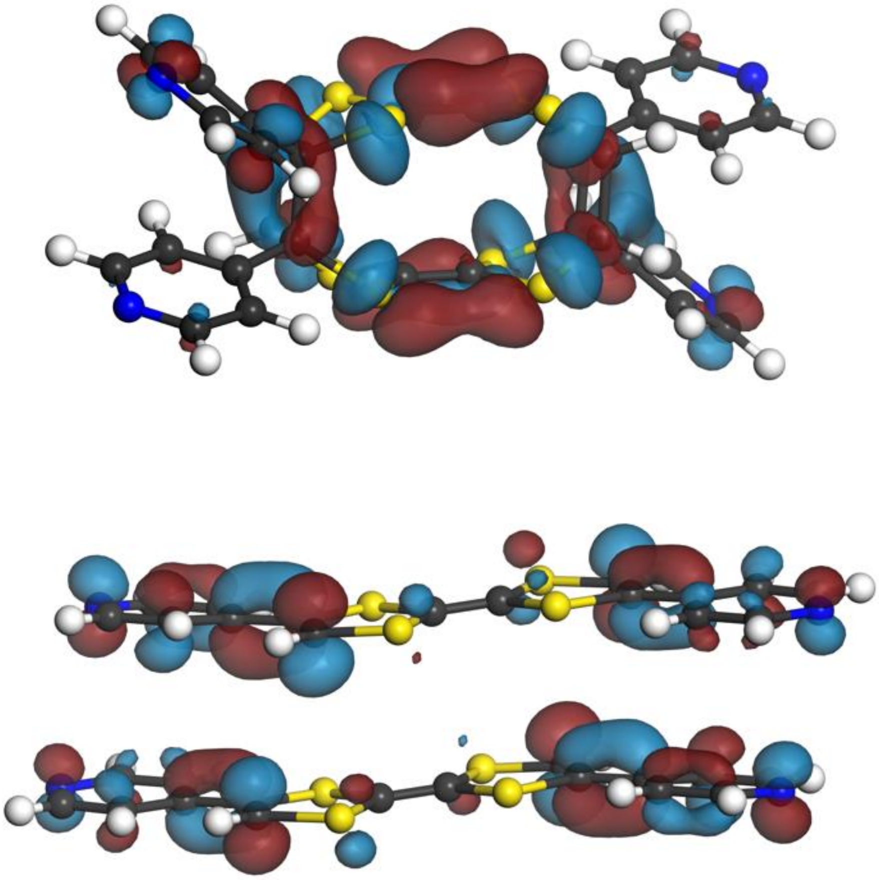

Supplementary Figure 10. Lowest Unoccupied Molecular Orbital (LUMO) of cyclised dimeric **Py_2TTF** (top) and cofacial dimeric **Py_2TTF** (bottom), not accounting for orbital interaction between the two dimers. Black = C, blue = N, yellow = S and white = H.

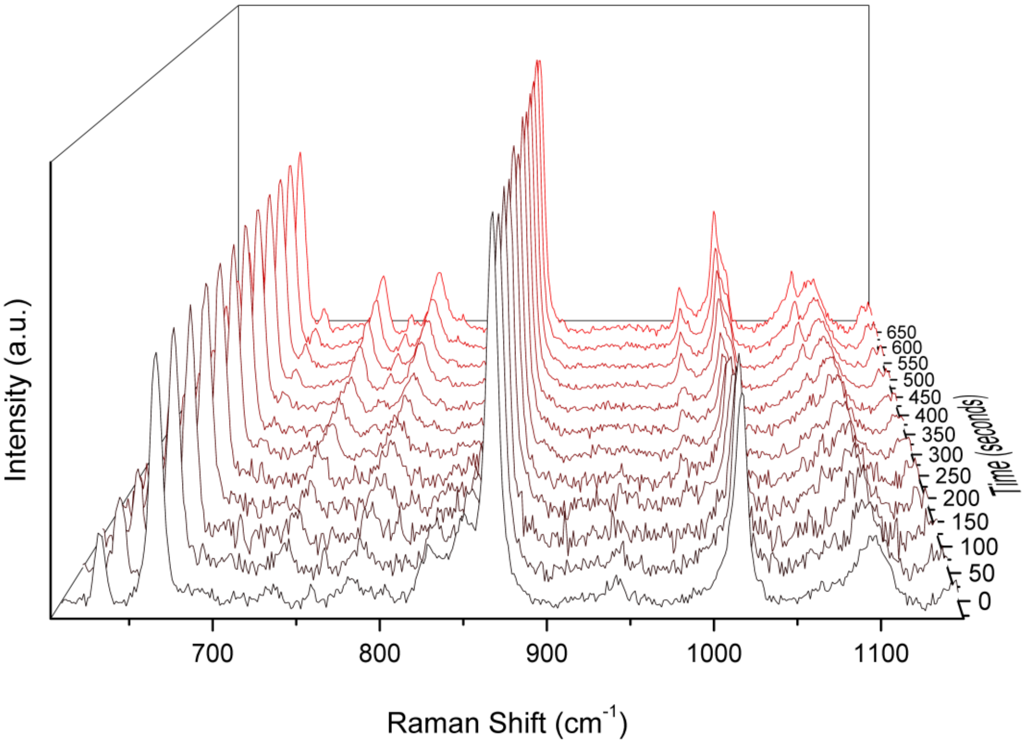

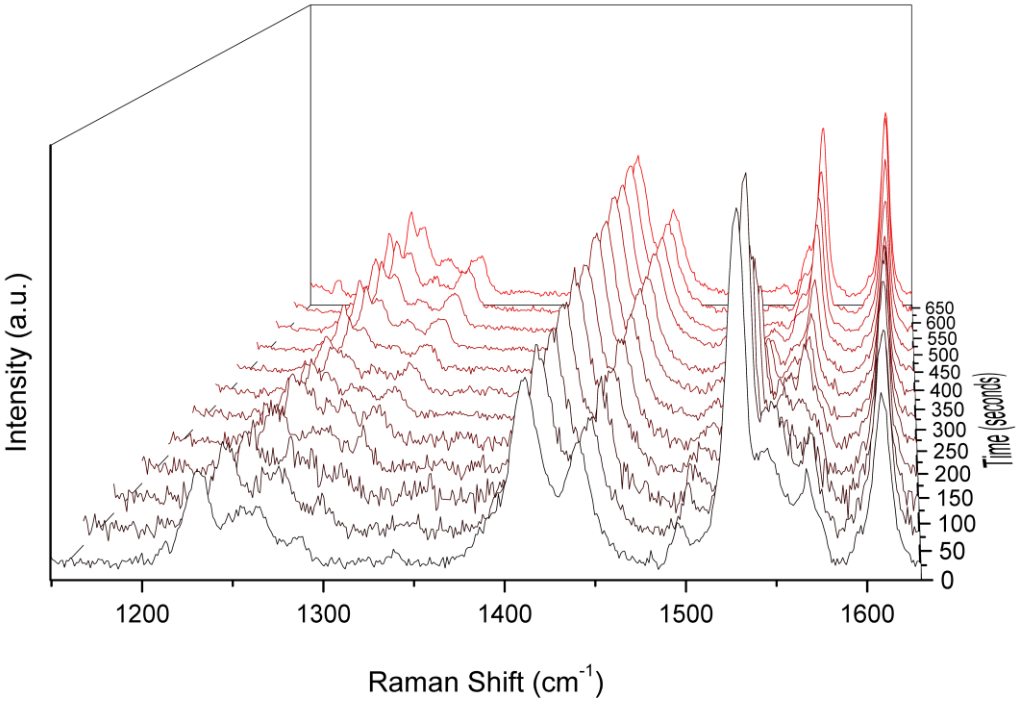

Supplementary Figure 11. Light-irradiated Raman (785 nm) of **1** between 600 to 1150 cm^{-1} (top) and 1150 to 1630 cm^{-1} (bottom). The first and the last spectra are shown in black (0 seconds) and red (630 seconds), respectively.

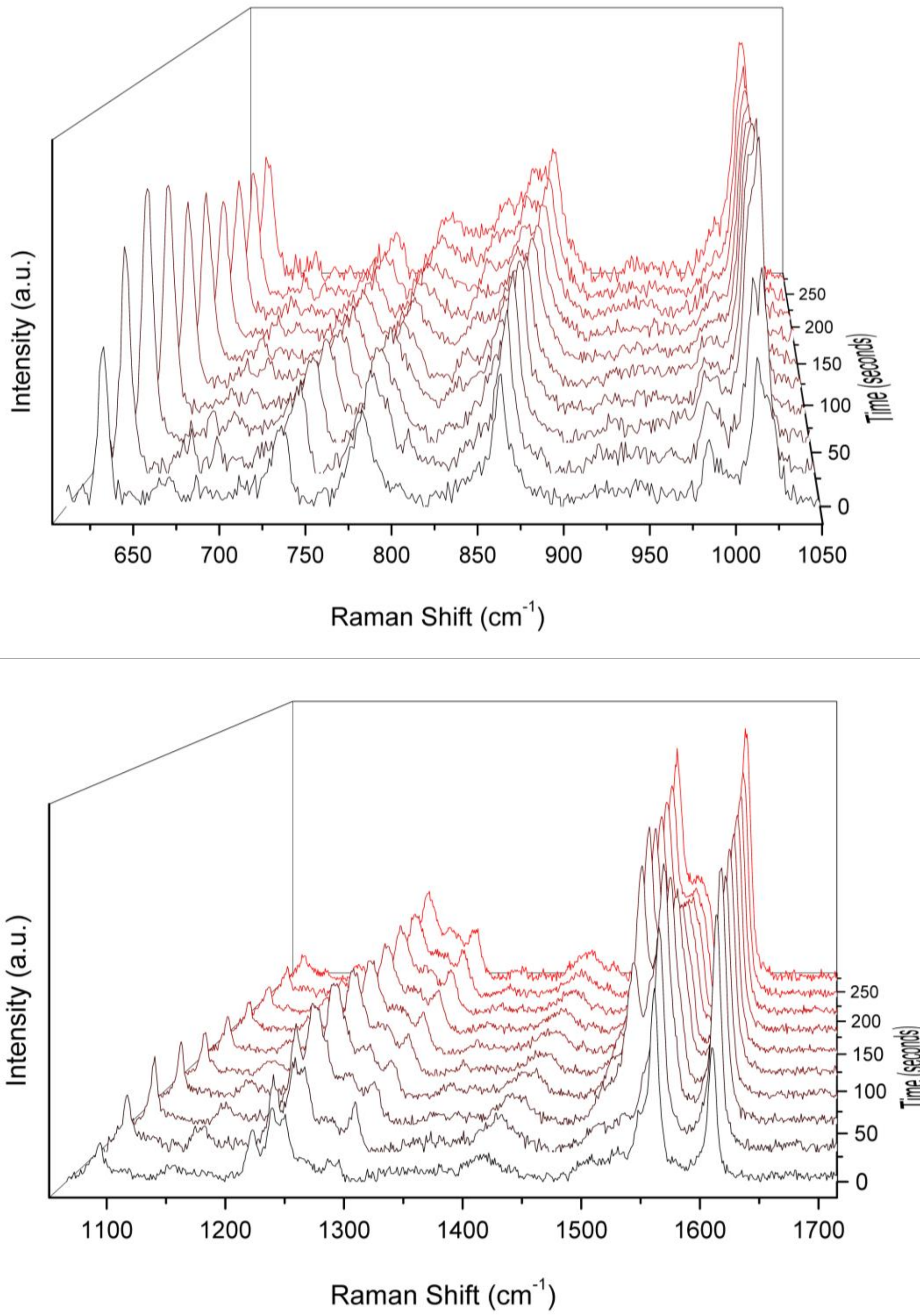

Supplementary Figure 12. Isothermal Raman (785 nm) of **3** at 180 °C between 550 to 1050 cm^{-1} (top) and 1050 to 1750 cm^{-1} (bottom). The first and the last spectra are shown in black (0 seconds) and red (660 seconds), respectively.

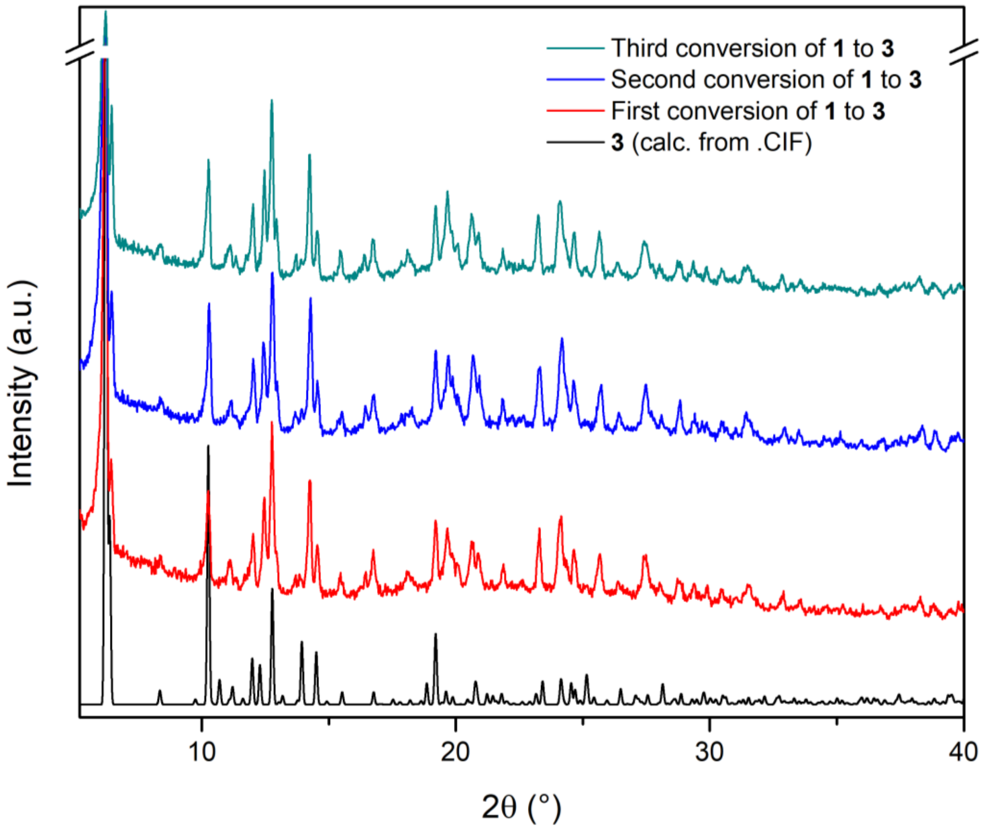

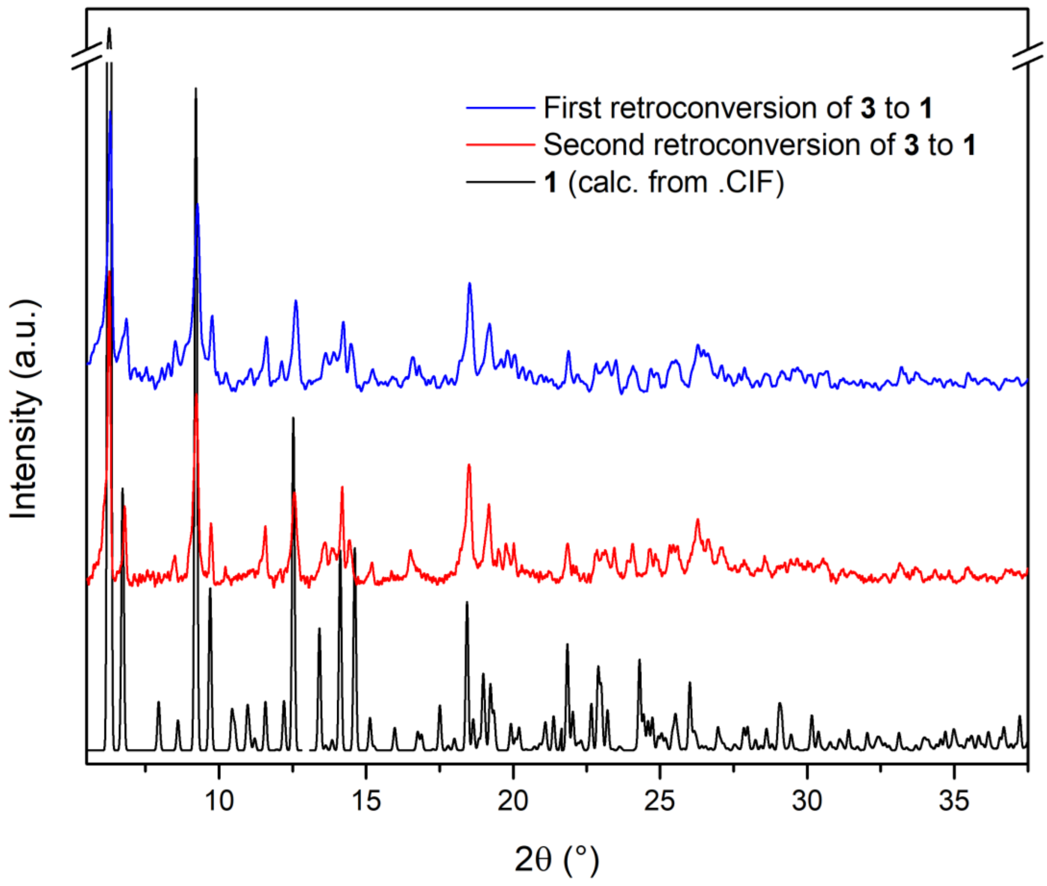

Supplementary Figure 13. Calculated PXRD patterns of **1** (bottom, black) and **3** (top, black); PXRD patterns of a sample of **1** converted to **3** (top, red), then retro-converted to **1** (bottom, blue), then re-converted to **3** (top, blue), then re-retro-converted to **1** (bottom, red), then converted a third time to **3** (top, green).

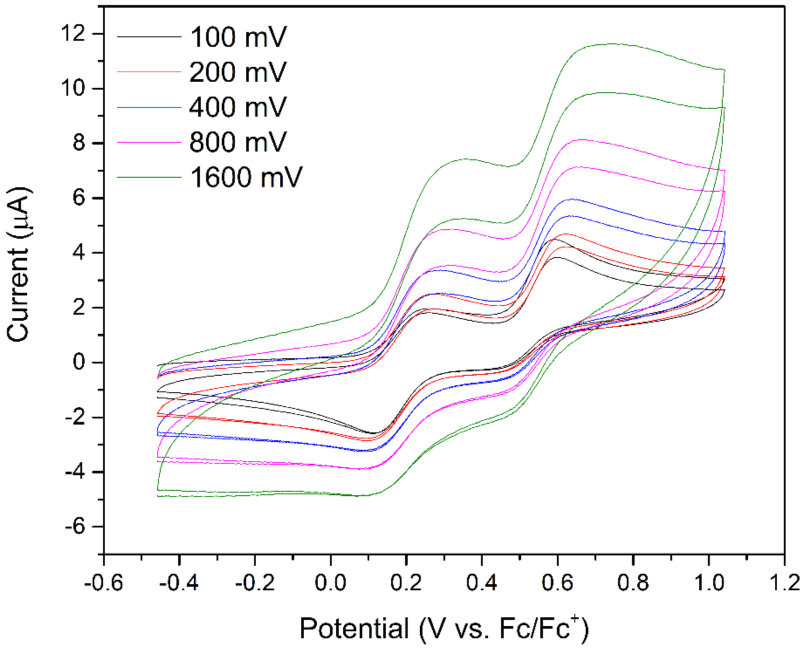

Supplementary Figure 14. Cyclic voltammogram of **1** in 0.1 M [(*n*-C_4H_9)$_4$N]PF_6/CH_3CN at various scan rates. Arrow indicates the direction of the forward scan.

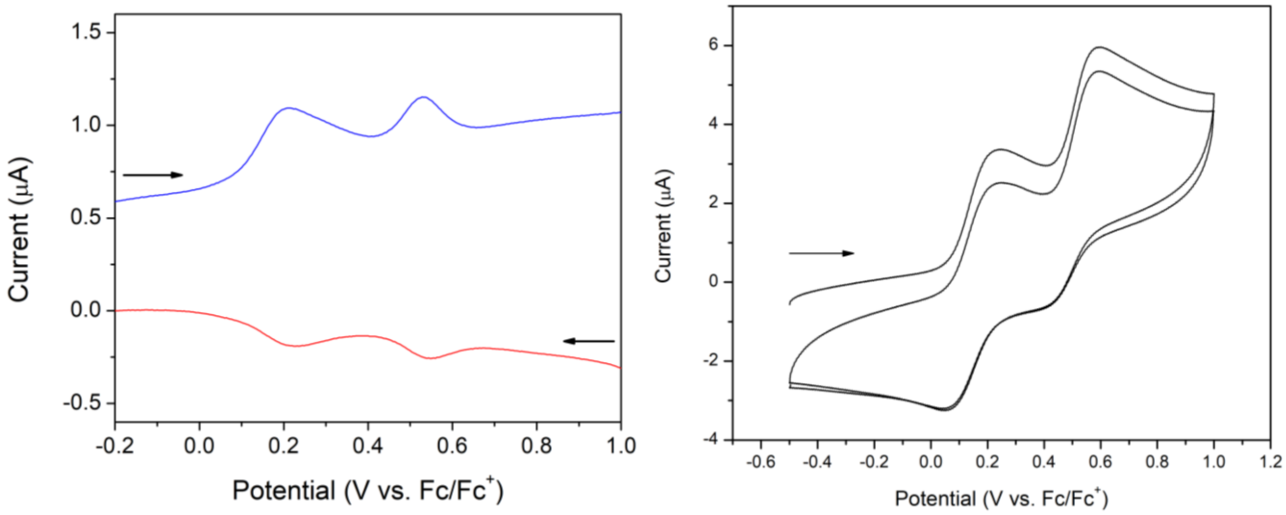

Supplementary Figure 15. Square wave voltammogram of a sample of **3** retro-converted to **1** in 0.1 M [(*n*-C_4H_9)$_4$N]PF_6/CH_3CN (left) and Cyclic voltammogram of a sample of **3** retro-converted to **1** in 0.1 M [(*n*-C_4H_9)$_4$N]PF_6/CH_3CN at 100 mV/s at scan rates of 100–1600 mV/s (right). Arrows indicate the direction of the forward scan.

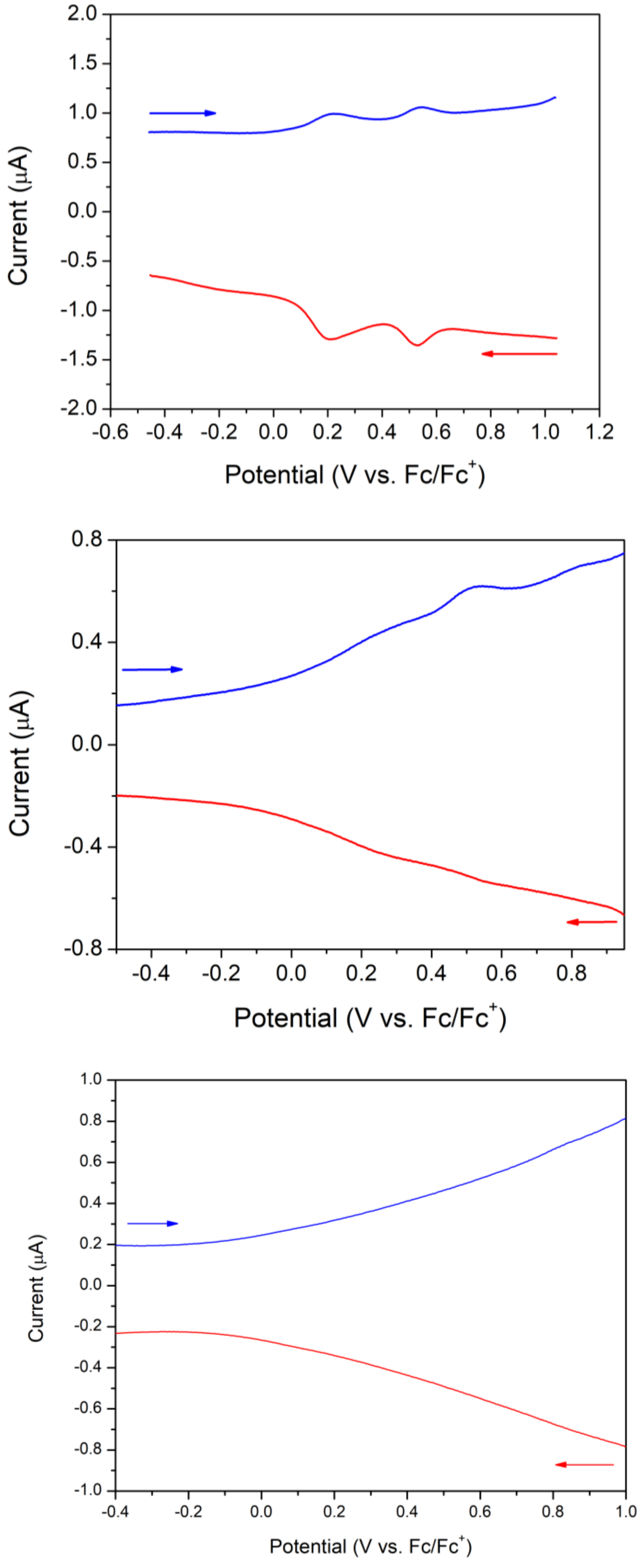

Supplementary Figure 16. Square wave voltammogram of **1** (top), **2** (middle) and **3** (bottom) in 0.1 M [(*n*-$C_4H_9)_4N]PF_6/CH_3CN$. Arrows indicate the direction of the forward scan.

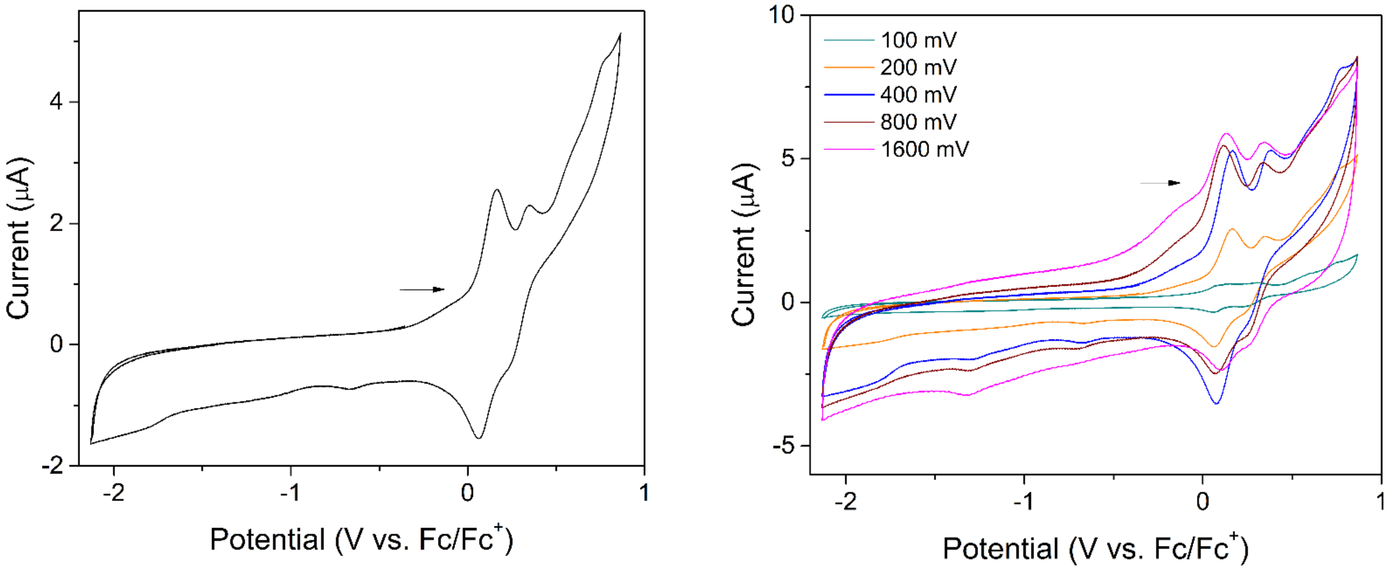

Supplementary Figure 17. Cyclic voltammogram of **4** in 0.1 M [(*n*-$C_4H_9)_4N]PF_6/CH_3CN$ at 100 mV/s (left) and at scan rates of 100–1600 mV/s (right). Arrows indicate the direction of the forward scan. The data shows the presence of two distinct *one*-electron processes at 0.12 and 0.30 V (*vs.* Fc/Fc^+), attributed to the oxidation of Py_2TTF to its radical cation, and dication, respectively.

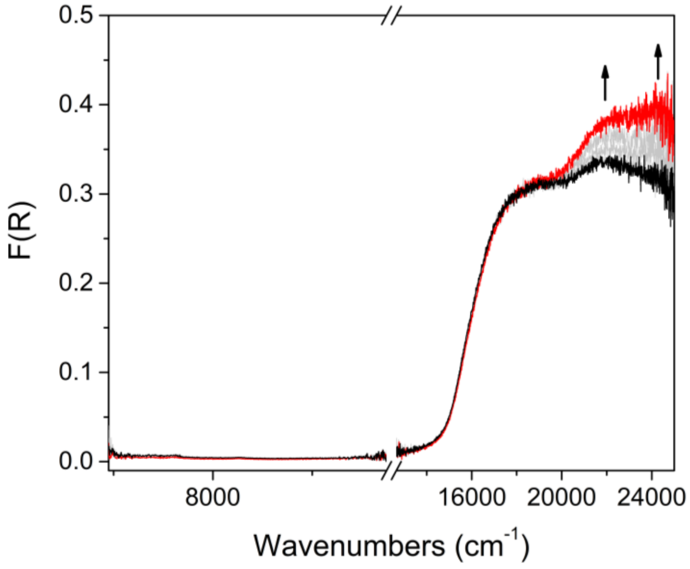

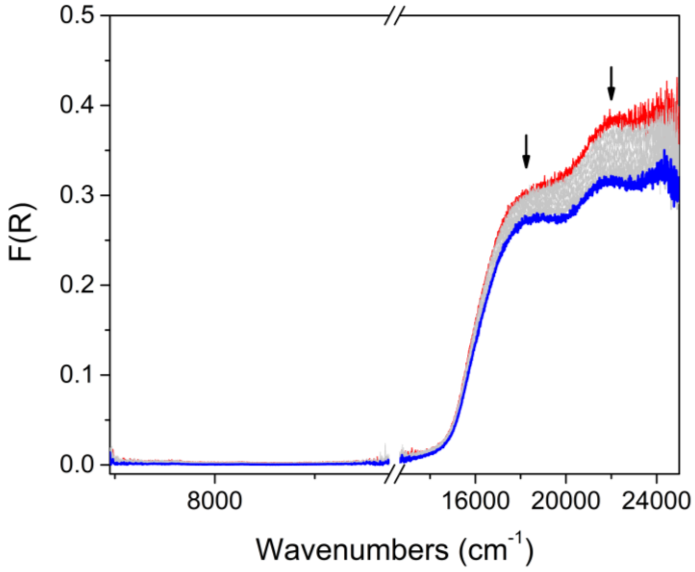

Supplementary Figure 18. Vis/NIR SEC of **4** in 0.1 M [(*n*-$C_4H_9)_4N]PF_6/CH_3CN$ between 0 to 0.2 V (left) and 0.2 to 0.7 V (right). Arrows indicate the direction of the spectral change. The first process which occurs between 0.0 to 0.2 V (*vs.* Ag/Ag^+) shows an intensification of the band at 22560 cm^{-1} (Figure S17). Increasing the potential to 0.7 V (*vs.* Ag/Ag^+) results in the decrease in the bands at 18570 and 22560 cm^{-1} due to oxidation of $Py_2TTF^{\cdot+}$ to Py_2TTF^{2+} (Figure S17). Notably, no changes were observed in the NIR region in our study. The absence of IVCT in this framework likely originates from the less favourable orientation and the longer distance between Py_2TTF units.

Supplementary References

1 Sheldrick, G. SHELXT - Integrated space-group and crystal-structure determination. *Acta Crystallographica Section A* **71**, 3-8, doi:0.1107/S2053273314026370 (2015).

2 Sheldrick, G. M. Crystal structure refinement with SHELXL. *Acta Crystallographica C* **71**, 3-8, doi:10.1107/S2053229614024218 (2015).

3 Usov, P. M., Fabian, C., D'Alessandro, D. M. Rapid determination of the optical and redox properties of a metal-organic framework via in situ solid state spectroelectrochemistry. *Chemical Communications* **48**, 3945-3947, doi:10.1039/c2cc30568b (2012).

4 Perdew, J. P., Wang, Y. Accurate and simple analytic representation of the electron-gas correlation energy. *Physical Review B* **45**, 13244-13249, doi:10.1103/PhysRevB.45.13244 (1992).

5 Frisch, M. J., *et al.* Gaussian 16, Revision A.03, Gaussian, Inc., Wallingford CT, 2016.

6 Han, Y.-F., Zhang, J.-S., Lin, Y.-J., Dai, J., Jin, G.-X. Synthesis and characterization of half-sandwich iridium complexes containing 2,6(7)-bis(4-pyridyl)-1,4,5,8-tetrathiafulvalene and ancillary ortho-carborane-1,2-dichalcogenolato ligands. *Journal of Organometallic Chemistry* **692**, 4545-4550 doi: 10.1016/j.jorganchem.2007.04.034 (2007).

7 Spek, A. L. PLATON SQUEEZE: a tool for the calculation of the disordered solvent contribution to the calculated structure factors. *Acta Crystallographica C* **71**, 9-18, doi:10.1107/S2053229614024929 (2015).

8 Spek, A. L. Structure validation in chemical crystallography. *Acta Crystallographica D* **65**, 148-155, doi:10.1107/S090744490804362X (2009).
